# Minimally invasive Ivor Lewis esophagectomy in the elderly patient: a multicenter retrospective matched-cohort study

**DOI:** 10.3389/fonc.2023.1104109

**Published:** 2023-05-12

**Authors:** Giovanni Capovilla, Eren Uzun, Alessia Scarton, Lucia Moletta, Edin Hadzijusufovic, Luca Provenzano, Renato Salvador, Elisa Sefora Pierobon, Gianpietro Zanchettin, Evangelos Tagkalos, Felix Berlth, Hauke Lang, Michele Valmasoni, Peter P. Grimminger

**Affiliations:** ^1^Department of Surgery, Oncology and Gastroenterology (DiSCOG), Padova University Hospital, Padova, Italy; ^2^Department of General, Visceral and Transplant Surgery, University Medical Center of the Johannes Gutenberg University, Mainz, Germany

**Keywords:** MIE, RAMIE, laparoscopy, thoracoscopy, esophagectomy, esophageal cancer

## Abstract

**Introduction:**

Several studies reported the advantages of minimally invasive esophagectomy over the conventional open approach, particularly in terms of postoperative morbidity and mortality. The literature regarding the elderly population is however scarce and it is still not clear whether elderly patients may benefit from a minimally invasive approach as the general population. We sought to evaluate whether thoracoscopic/ laparoscopic (MIE) or fully robotic (RAMIE) Ivor-Lewis esophagectomy significantly reduces postoperative morbidity in the elderly population.

**Methods:**

We analyzed data of patients who underwent open esophagectomy or MIE/RAMIE at Mainz University Hospital and at Padova University Hospital between 2016 and 2021. Elderly patients were defined as those ≥ 75 years old. Clinical characteristics and the postoperative outcomes were compared between elderly patients who underwent open esophagectomy or MIE/RAMIE. A 1-to-1 matched comparison was also performed. Patients < 75 years old were evaluated as a control group.

**Results:**

Among elderly patients MIE/RAMIE were associated with a lower overall morbidity (39.7% vs. 62.7%, p=0.005), less pulmonary complications (32.8 vs. 56.9%, p=0.003) and a shorter hospital stay (13 vs. 18 days, p=0.03). Comparable findings were obtained after matching. Similarly, among < 75 years-old patients, a reduced morbidity (31.2% vs. 43.5%, p=0.01) and less pulmonary complications (22% vs. 36%, p=0.001) were detected in the minimally invasive group.

**Discussion:**

Minimally invasive esophagectomy improves the postoperative course of elderly patients reducing the overall incidence of postoperative complications, particularly of pulmonary complications.

## 1 Introduction

Ivor Lewis esophagectomy is a complex procedure, burdened by a high rate of postoperative complications and mortality (1–3). The use of a minimally invasive approach has been associated with better perioperative outcomes, however most of the published studies are conducted on the general population (4–6) and the literature evaluating the outcomes in elderly patients is rather limited (7–10). Furthermore, most of the available data focus on the differences in the postoperative outcomes between groups of elderly and non-elderly patients (7–9), while the evaluation of the benefits provided by a minimally invasive approach compared to open surgery within the different age groups is seldom performed (10). Elderly subjects represent indeed a fragile subset, often presenting in worse clinical conditions and with a poor performance status. It is therefore not clear whether the same improvement in the postoperative course seen in the general population undergoing minimally invasive esophagectomy could be expected in older individuals.

Aim of our study was to evaluate the short-term postoperative outcome of minimally invasive Ivor Lewis esophagectomy and to assess whether the use of this approach provides the same improvement in the postoperative course for both elderly and non-elderly patients.

## 2 Methods

### 2.1 Study population

We retrospectively reviewed prospectively collected records from 2016 to 2021 of all patients with esophageal cancer who referred to two high volume centers for upper-GI surgery: Mainz University Hospital (Germany) and Padova University Hospital (Italy) and underwent Ivor-Lewis esophagectomy with either an open or a laparoscopic/thoracoscopic approach (minimally invasive esophagectomy, MIE) or a fully robotic approach (robotic-assisted minimally invasive esophagectomy, RAMIE).

Only patients with esophageal squamous cell carcinoma (ESCC) or adenocarcinoma (EAC) were considered recruitable. Patients with cT4b or M+ disease, patients with cervical or Siewert 3 cancers and those who underwent R2, or palliative resections were excluded from the study. Patients who underwent upfront surgery or multimodal treatment comprising chemotherapy (CT) and/or radiotherapy (RT) and surgery were recruited.

### 2.2 Study design

Elderly patients were defined as those who were ≥ 75 years old at the time of surgery (≥ 75y group) and represented the study group. The control group consisted of patients being < 75 years old at surgery (< 75y group). Differences in the clinical characteristics of the two groups were compared by univariate analysis. A subset analysis of the ≥ 75y group was performed comparing the clinical characteristics and the postoperative outcome of elderly patients who underwent open esophagectomy (≥ 75y open group) and MIE/RAMIE (≥ 75y MI group). The same analysis was performed within the control group (< 75y open group vs. < 75y MI group).

The univariate analysis of preoperative and postoperative outcomes was then repeated within the ≥ 75y group after one-to-one matching between the ≥ 75y open group and the ≥ 75y MI group. The two subgroups were matched for the following potential confounding factors: age, sex, ASA score, cancer histology, cancer location and preoperative treatment. Primary outcome of the study were the short-term post-surgical morbidity and mortality.

### 2.3 Collected data and definitions

EAC and ESCC were graded according to AJCC 8^th^ edition Classification (11). The overall patients’ preoperative condition was assessed using the Karnofsky performance status (KPS) (12); the operative risk was evaluated using the American Society of Anestesiology (ASA) classification (13) and the Charlson’s Comorbidity Index (CCI) (14).

Post-operative 90-day complications were assessed according to the Esophagectomy Complications Consensus Group ECCG (15) and graded according to the Clavien-Dindo classification (16).

### 2.4 Clinical staging and preoperative treatment

Esophagogastroduodenoscopy with biopsy, CT scan of the cervical, thoracic, and abdominal regions were used for the clinical staging. The evaluation was completed using positron emission tomography (PET/CT) and endoscopic ultrasound (EUS) when deemed necessary. Bronchoscopy was performed in all patients with SCC and in those with possible airways infiltration. The neoadjuvant treatment was not standardized as patients were frequently referred for surgery after being treated at other centers, therefore variations in the chosen regimens could occur based on the preferences of the treating oncologists or the patients’ conditions and comorbidities. However, perioperative chemotherapy with FLOT (17) or preoperative chemoradiotherapy with the CROSS scheme (18) were the most frequently used regimens.

### 2.5 Surgical technique

The surgical techniques used for open esophagectomy (19–21), MIE (22, 23) and RAMIE (24, 25) were previously described. Briefly, all procedures included the mobilization of the stomach and the creation of a gastric conduit in the abdominal part. In the thoracic part, the esophagus was mobilized and transected above the azygos vein. A standard two-field lymphadenectomy was performed (26). The anastomosis was secured using an end-to-side circular-stapled technique. For the open procedure a median laparotomy and a posterolateral or anterolateral right thoracotomy were performed, with the patient in a left lateral decubitus during the thoracic phase. For both MIE and RAMIE, after the abdominal phase the patient was placed in the semi-prone position and the thoracic phase was performed with 4 operative trocars placed along the anterior axillary line (one additional assistant-trocar was used for RAMIE).

All patients were intubated using a left-sided double-lumen tube and a one-lung ventilation was used throughout the thoracic phase. Analgesia was provided by means of an epidural catheter. Postoperative care included early extubation, preferably in the operatory room, epidural- and patient-controlled analgesia, respiratory exercise, and early mobilization and ambulation. After surgery, patients were admitted to the intensive care unit (ICU) and subsequently discharged towards the surgical ward upon confirmation of the hemodynamical and respiratory stability. No enhanced recovery program was used.

### 2.6 Statistical analysis

All statistical analyses were conducted using GraphPad Prism version 9.2.0 (GraphPad software, San Diego, CA, USA) and JMP version 14 (JMP^®^ software, SAS Institute, Cary, NC, USA). Continuous variables were presented as median (interquartile range [IQR]), prevalence data were presented as raw number (percentage). Comparisons of continuous variables were conducted using the Student’s t-test or the Mann-Whitney test. ANOVA or Kruskal Wallis tests were used for multiple comparisons of continuous variables as appropriate. Shapiro-Wilk test was applied to test the normality of the data (p > 0.10). Categorical data were compared using the χ^2^ or the Fisher’s exact test as appropriate. The Bonferroni correction for multiple comparisons was applied when indicated. The threshold for statistical significance was set to p < 0.05. For the purpose of randomization, a one-to-one nearest neighbor approach was used for the selection of patients in the matched control group.

## 3 Results

Clinical characteristics of the studied population are summarized in **Table 1**. Elderly patients presented with a higher comorbidity index and with a worse physical- (p < 0.0001) and performance-status (p = 0.003). EAC was the most frequent histology, however a significantly higher proportion of patients in the ≥ 75y group presented with ESCCs (p = 0.0007) located in the mid-lower portion of the thoracic esophagus (p = 0.0003). Despite no significant difference in the cancer stage at presentation (p = 0.92), multimodal treatment comprising chemotherapy or chemoradiotherapy before surgery was less frequently used in elderly patients (p < 0.0001) and an open approach rather than a minimally invasive one was preferred in this subgroup (p <0.0001).

**Table 1:** Clinical characteristic of the studied population.

Variable	< 75y group (N=427)	≥ 75y group (N=160)	p
Median age (years)	61 (55-67)	78 (76-81)	< 0.0001
Sex (N, %)			
Male	356 (83.4)	119 (74.4)	0.01
Female	71 (16.6)	41 (25.6)	
Height (m)	1.76 (1.70-1.80)	1.72 (1.67-1.78)	0.04
Weight (kg)	77 (67.5-88)	74 (65-85.5)	0.12
ASA score (N, %)			
1	5 (1.2)	1 (0.6)	< 0.0001
2	222 (52)	41 (25.6)	
3	194 (45.4)	98 (61.3)	
4	6 (1.4)	20 (12.5)	
Karnofsky performance status (N, %)*			
100-90	380 (89)	125 (78.1)	0.003
80-70	44 (10.3)	33 (20.6)	
60-50	3 (0.7)	2 (1.3)	
Charlson’s Comorbidity Index (CCI)	4 (3-5)	6 (5-7)	< 0.0001
Cancer histology (N, %)			
ESCC	78 (18.3)	50 (31.3)	0.0007
EAC	349 (81.7)	110 (68.7)	
Cancer location (N, %)			
Thoracic esophagus	52 (12.2)	41 (25.6)	0.0003
Siewert 1	212 (49.6)	71 (44.4)	
Siewert 2	163 (38.2)	48 (30)	
cTNM Staging			
Stage 1	39 (9.1)	13 (8.1)	0.92
Stage 2	88 (20.6)	31 (19.4)	
Stage 3	273 (63.9)	104 (65)	
Stage 4	27 (6.4)	12 (7.5)	
Perioperative treatment (N, %)			
None	67 (15.7)	96 (60)	< 0.0001
Chemotherapy	168 (39.3)	33 (20.6)	
Chemoradiotherapy	190 (44.5)	29 (18.1)	
Radiotherapy	2 (0.5)	2 (1.3)	
Surgical approach (N, %)			
Open	178 (41.7)	102 (63.7)	< 0.0001
Laparoscopic/Thoracoscopic (MIE)	94 (22)	24 (15)	
Fully Robotic (RAMIE)	155 (36.3)	34 (21.3)	

Subset analyses of the < 75y group and the ≥ 75y group are reported in **Table 2**. Within the < 75y group no difference was detected in the patients’ clinical characteristics at presentation. Albeit not statistically significant, a higher proportion of patients in the < 75y MI group presented with ESCC (21.2% vs. 14.1%, p = 0.07). Among elderly patients, the male sex was prevalent, however, a significantly higher proportion of patients in the ≥ 75y open group was female (31.4% vs. 15.5%, p = 0.03). No further difference was detected in the preoperative characteristics of these patients.

**Table 2:** Clinical characteristics of the analyzed subgroups.

Variable	< 75y MI group (N=250)	< 75y open group (N=177)	p	≥ 75y MI group (N=58)	≥ 75y open group (N=102)	p
Median age (years)	61 (55-68)	60 (53-67)	0.28	79 (76-82)	77 (76-80)	0.51
Sex (N, %)						
Male	205 (82)	151 (85.3)	0.37	49 (84.5)	70 (68.6)	0.03
Female	45 (18)	26 (14.7)		9 (15.5)	32 (31.4)	
Height (m)	1.76 (1.65-1.79)	1.77 (1.71-1.80)	0.42	1.72 (1.67-1.78)	1.70 (1.58-1.75)	0.28
Weight (kg)	77 (68-88)	79 (69-91)	0.33	74 (65.5-86)	72 (59-81.5)	0.53
ASA score (N, %)						
1	3 (1.2)	2 (1.1)	0.13	0	1 (0.9)	0.87
2	119 (47.6)	103 (58.2)		15 (25.9)	26 (25.5)	
3	123 (49.2)	71 (40.1)		35 (60.3)	63 (61.8)	
4	5 (2)	1 (0.6)		8 (13.8)	12 (11.8)	
Karnofsky performance status (N, %)						
100-90	219 (87.6)	161 (91)	0.26	45 (77.6)	80 (78.5)	0.92
80-70	28 (11.2)	16 (9)		12 (20.7)	21 (20.6)	
60-50	3 (1.2)	0		1 (1.7)	1 (0.9)	
Charlson’s Comorbidity Index (CCI)	5 (3.5-6.5)	4 (3-5)	0.15	5 (3-7)	6 (5-7)	0.28
Cancer histology (N, %)						
ESCC	53 (21.2)	25 (14.1)	0.07	18 (31)	32 (31.4)	0.96
EAC	197 (78.8)	152 (85.9)		40 (69)	70 (68.6)	
Cancer location (N, %)						
Thoracic esophagus	30 (12)	22 (12.4)	0.37	11 (19)	30 (29.4)	0.25
Siewert 1	131 (52.4)	81 (45.8)		26 (44.8)	45 (44.1)	
Siewert 2	89 (35.6)	74 (41.8)		21 (36.2)	27 (26.5)	
cTNM Staging						
Stage 1	29 (11.6)	10 (5.6)	0.17	6 (10.3)	7 (6.9)	0.71
Stage 2	51 (20.4)	37 (20.9)		9 (15.5)	22 (21.6)	
Stage 3	153 (61.2)	120 (67.8)		39 (67.2)	65 (63.7)	
Stage 4	17 (6.8)	10 (5.6)		4 (7)	8 (7.8)	
Perioperative treatment (N, %)						
None	43 (17.2)	24 (13.6)	0.40	31 (53.5)	65 (63.8)	0.29
Chemotherapy	103 (41.2)	65 (36.7)		13 (22.4)	20 (19.6)	
Chemoradiotherapy	103 (41.2)	87 (49.1)		14 (24.1)	15 (14.7)	
Radiotherapy	1 (0.4)	1 (0.6)		0	2 (1.9)	
Surgical approach (N, %)						
Laparoscopic/Thoracoscopic (MIE)	95 (38)	–	–	24 (41.4)	–	–
Fully Robotic (RAMIE)	155 (62)	–		34 (58.6)	–	
Operative time (min)	331 (289-380)	285 (245-331)	0.02	340 (310-377)	298 (243-334)	0.03

“-” means the variable in the row is not applicable to the patients in the column.

Surgical outcomes are depicted in **Table 3**. The use of a minimally invasive approach in elderly patients did not compromise the surgical radicality of the primary tumor resection (p = 0.99) and of the lymph nodes dissection (p = 0.53). These results were paralleled by those of the control group. Overall, the proportion of patients experiencing postoperative complications was significantly higher in the ≥ 75y open group compared to the ≥ 75y MI group (62.7% vs. 39.7%, p = 0.005). The main determinant of the increased morbidity in the ≥ 75y open group were pulmonary complications, which increased significantly after open surgery (56.9% vs. 32.8%, p = 0.003) and consisted mainly of pneumonia (20.6% vs. 8.6%, p = 0.04) and mucous plugging requiring bronchoscopy (22.6% vs. 10.3%, p = 0.05). Similar results were obtained in the < 75y group: postoperative morbidity (43.5% vs. 31.2%, p = 0.01) and pulmonary complication (36% vs. 22%, p = 0.001) were significantly higher after open surgery. Pneumonia (11.9% vs. 6%, p = 0.03) and mucous plugging (12.4% vs. 7.2%, p = 0.04) were the most frequently reported pulmonary complications also in this subgroup.

**Table 3:** Surgical outcomes of the analyzed subgroups.

Variable	< 75y MI group (N=250)	< 75y open group (N=177)	p	≥ 75y MI group (N=58)	≥ 75y open group (N=102)	p
Surgical radicality (N, %)						
R0	245	173	0.99	56	99	0.99
R1	5	4		2	3	
Harvested lymph nodes (N, IQR)	31 (23-40)	28 (20-35)	0.68	27 (19-33)	25 (17-31)	0.53
Metastatic lymph nodes (N, IQR)	2 (0-3)	2 (0-3)	0.28	2 (0-3)	1 (0-4)	0.33
Intraoperative complications (N, %)	8 (3.2)	5 (2.8)	0.82	4 (6.9)	5 (4.9)	0.72
Postoperative morbidity (N, %)	78 (31.2)	77 (43.5)	0.01	23 (39.7)	64 (62.7)	0.005
Anastomotic leakage (N, %)	26 (10.4)	15 (8.4)	0.51	7 (12)	15 (14.6)	0.64
Grade 1	2 (0.8)	1 (0.5)	0.97	1 (1.7)	1 (0.9)	0.83
Grade 2	20 (8)	12 (6.8)		5 (8.6)	12 (11.8)	
Grade 3	4 (1.6)	2 (1.1)		1 (1.7)	2 (1.9)	
Conduit necrosis (N, %)	1 (0.4)	2 (1.1)	0.57	1 (1.7)	2 (1.9)	0.99
Chyle leak (N, %)	8 (3.2)	3 (1.7)	0.54	3 (5.2)	4 (3.9)	0.70
Vocal cord palsy (N, %)	3 (1.2)	2 (1.1)	0.99	0	4 (3.9)	0.29
Hemothorax (N,%)	8 (3.2)	12 (6.8)	0.08	1 (1.7)	6 (5.9)	0.42
Pulmonary complications (N,%)*	55 (22)	64 (36)	0.001	19 (32.8)	58 (56.9)	0.003
Pneumonia	15 (6)	21 (11.9)	0.03	5 (8.6)	21 (20.6)	0.04
Atelectasis mucous plugging requiring bronchoscopy	18 (7.2)	22 (12.4)	0.04	6 (10.3)	23 (22.6)	0.05
Pneumothorax	7 (2.8)	7 (3.9)	0.59	1 (1.7)	4 (3.9)	0.65
Pleural effusion requiring drainage	7 (2.8)	5 (2.8)	0.99	2 (3.4)	5 (4.9)	0.99
Respiratory failure requiring reintubation	5 (2)	6 (3.4)	0.37	3 (5.2)	4 (3.9)	0.70
ARDS	3 (1.2)	3 (1.7)	0.69	0	1 (0.9)	0.99
Cardiac complications (N,%)*	19 (7.6)	17 (9.4)	0.46	6 (10.3)	10 (9.8)	0.91
Atrial dysrhythmia requiring treatment	11 (4.4)	12 (6.8)	0.28	4 (6.9)	5 (4.9)	0.72
Congestive heart failure requiring treatment	8 (3.2)	5 (2.8)	0.99	2 (3.4)	4 (3.9)	0.99
Myocardial infarction	0	0	0.99	0	1 (0.9)	0.99
Gastrointestinal complications (N,%)*	7 (2.8)	9 (5.1)	0.22	1 (1.7)	4 (3.9)	0.65
*Clostridium difficile* infection	1 (0.4)	2 (1.1)	0.57	1 (1.7)	3 (2.9)	0.99
Liver dysfunction	1 (0.4)	1 (0.5)	0.99	0	1 (0.9)	0.99
Sepsis	5 (2)	6 (3.4)	0.37	2 (3.4)	11 (10.8)	0.10
Complications: severity^b^ (N, %)						
1	6 (2.4)	8 (4.5)	0.88	1 (1.7)	3 (2.9)	0.87
2	23 (9.2)	20 (11.3)		5 (8.6)	10 (9.8)	
3a	19 (7.6)	16 (9)		6 (8.6)	26 (25.5)	
3b	11 (4.4)	12 (6.8)		3 (5.2)	8 (7.8)	
4	14 (5.6)	18 (10.2)		6 (8.6)	12 (11.8)	
5	5 (2)	3 (1.6)		2 (3.4)	5 (4.9)	
Need for reoperation (N, %)	15 (6)	16 (9)	0.23	4 (6.9)	11 (10.8)	0.43
ICU readmission (N, %)	18 (7.2)	21 (11.9)	0.09	7 (12.1)	17 (16.7)	0.49
Length of hospital stay (days)(N, IQR)	12 (10-17)	12 (10-18)	0.19	13 (11-19)	18 (13-26)	0.03
In-hospital mortality (N, %)	5 (2)	3 (1.6)	0.99	2 (3.4)	5 (4.9)	0.99

*The most severe complication for each category is indicated.

^b^ According to the Clavien-Dindo classification.

The incidence and severity of anastomotic leakage did not differ after MI and open surgery within both the < 75y group and ≥ 75y group. Grade 2 leakages were primarily treated by an endoscopically placed nasogastric tube, vacuum-device (EsoSponge^®^, B. Braun, Melsungen, Germany) or stent and did not require a reoperation. Grade 3 leakages required reoperation with dismantling of the gastric pull-up and cervical esophagostomy.

Overall, 15 patients required reoperation in the ≥ 75y group (9.4%), the main reasons were the presence of a grade 3 leakage (3 patients) or conduit necrosis (3 patients), hemothorax (6 patients), chyle leak (1 patient) and postoperative abdominal bleeding (2 patients). Thirty-one patients required a reoperation in the < 75y group (7.3%). The main causes of reoperation were grade 3 leakage (6 patients) or conduit necrosis (3 patients), hemothorax (16 patients), chyle leak (2 patients), bowel perforation (1 patient) and abdominal bleeding (2 patients). The rate of reoperations, the severity of postoperative complications (Clavien Dindo grade) and the rate of in-hospital mortality were similar after MI or open surgery in both study groups. Patients of the ≥ 75y open group required a longer hospital stay compared to the ≥ 75y MI group (p = 0.03).

**Table 4** reports the clinical characteristics of the ≥ 75y MI group and the ≥ 75y open group after one-to-one matching for patient’s sex, cancer stage and preoperative treatment. The matching resulted in no significant difference in the preoperative variables.

**Table 4:** Clinical characteristics of the analyzed subgroups after matching.

Variable	≥ 75y MI group (N=58)	≥ 75y open group (N=58)	p
Median age (years)	79 (76-82)	78 (75-82)	0.43
Sex (N, %)			
Male	49 (84.5)	50 (86.2)	0.79
Female	9 (15.5)	8 (13.8)	
Height (m)	1.72 (1.67-1.78)	1.71 (1.60-1.76)	0.16
Weight (kg)	74 (65.5-86)	70 (55-78.3)	0.12
ASA score (N, %)			
1	0	1 (1.7)	0.74
2	15 (25.9)	13 (22.4)	
3	35 (60.3)	37 (63.8)	
4	8 (13.8)	7 (12.1)	
Karnofsky performance status (N, %)			
100-90	45 (77.6)	45 (77.6)	0.59
80-70	12 (20.7)	13 (22.4)	
60-50	1 (1.7)	0	
Charlson’s Comorbidity Index (CCI)	5 (3-7)	5 (4-7)	0.72
Cancer histology (N, %)			
ESCC	18 (31)	19 (32.8)	0.84
EAC	40 (69)	39 (67.2)	
Cancer location (N, %)			
Thoracic esophagus	11 (19)	13 (22.4)	0.90
Siewert 1	26 (44.8)	25 (43.1)	
Siewert 2	21 (36.2)	20 (34.5)	
cTNM Staging			
Stage 1	6 (10.3)	7 (12.1)	0.75
Stage 2	9 (15.5)	12 (20.7)	
Stage 3	39 (67.2)	37 (63.8)	
Stage 4	4 (7)	2 (3.4)	
Perioperative treatment (N, %)			
None	31 (53.5)	29 (50)	0.81
Chemotherapy	13 (22.4)	16 (27.6)	
Chemoradiotherapy	14 (24.1)	13 (22.4)	
Radiotherapy	0	0	

**Table 5** summarizes the postoperative outcomes of the two subgroups after the matching. The matched analysis confirmed a significantly higher rate of postoperative complications in the ≥ 75y open group (62.1% vs. 39.7%, p = 0.02) with pulmonary complications (p = 0.04) being the main determinant of the postoperative morbidity.

**Table 5:** Surgical outcomes of the analyzed subgroups after matching.

Variable	≥ 75y MI group (N=58)	≥ 75y open group (N=58)	p
Surgical radicality (N, %)			
R0	56	57	0.99
R1	2	1	
Harvested lymph nodes (N, IQR)	27 (19-33)	26 (16-32)	0.48
Metastatic lymph nodes (N, IQR)	2 (0-3)	1 (0-4)	0.60
Intraoperative complications (N, %)	4 (6.9)	2 (3.4)	0.68
Postoperative morbidity (N, %)	23 (39.7)	36 (62.1)	0.02
Anastomotic leakage (N, %)	7 (12.1)	5 (8.6)	0.76
Grade 1	1 (1.7)	1 (1.7)	0.92
Grade 2	5 (8.6)	3 (5.2)	
Grade 3	1 (1.7)	1 (1.7)	
Conduit necrosis (N, %)	1 (1.7)	0	0.99
Chyle leak (N, %)	3 (5.2)	1 (1.7)	0.62
Vocal cord palsy (N, %)	0	1 (1.7)	0.99
Hemothorax (N,%)	1 (1.7)	4 (6.9)	0.36
Pulmonary complications (N,%)*	19 (32.8)	30 (51.7)	0.04
Pneumonia	5 (8.6)	12 (20.7)	0.07
Atelectasis mucous plugging requiring bronchoscopy	6 (10.3)	15 (25.9)	0.03
Pneumothorax	1 (1.7)	0	0.99
Pleural effusion requiring drainage	2 (3.4)	2 (3.4)	0.99
Respiratory failure requiring reintubation	3 (5.2)	1 (1.7)	0.62
ARDS	0	0	0.99
Cardiac complications (N,%)*	6 (10.3)	5 (8.6)	0.99
Atrial dysrhythmia requiring treatment	4 (6.9)	3 (5.2)	0.99
Congestive heart failure requiring treatment	2 (3.4)	2 (3.4)	0.99
Myocardial infarction	0	0	0.99
Gastrointestinal complications (N,%)*	1 (1.7)	1 (1.7)	0.99
*Clostridium difficile* infection	1 (1.7)	1 (1.7)	0.99
Liver dysfunction	0	0	0.99
Sepsis	2 (3.4)	6 (10.3)	0.27
Complications: severity^b^ (N, %)			
1	1 (1.7)	1 (1.7)	0.92
2	5 (8.6)	9 (15.5)	
3a	6 (8.6)	12 (20.7)	
3b	3 (5.2)	4 (6.9)	
4	6 (8.6)	9 (15.5)	
5	2 (3.4)	1 (1.7)	
Need for reoperation (N, %)	4 (6.9)	6 (10.3)	0.51
ICU readmission (N, %)	7 (12.1)	10 (17.2)	0.43
Length of hospital stay (days)(N, IQR)	13 (11-19)	15 (11-20)	0.09
In-hospital mortality (N, %)	2 (3.4)	1 (1.7)	0.99

* The most severe complication for each cathegory is indicated.

^b^ According to the Clavien-Dindo classification.

**Figure 1:**
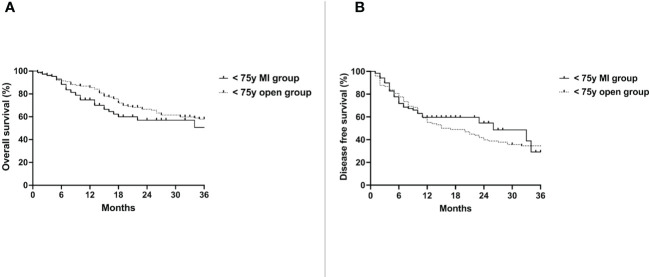
Overall survival **(A)** and disease free survival **(B)** of the < 75y MI group vs. the < 75y open group. Whole cohort.

Median follow up time was 28 months (10-84). Median overall survival (OS) of the < 75y MI group was 62 months and 49 months in the < 75y open group (p=0.35) (**Figure 1A**). The disease free survival (DFS) of the two groups was also not significantly different (26 vs. 16 months, p=0.59) (**Figure 1B**).

**Figure 2:**
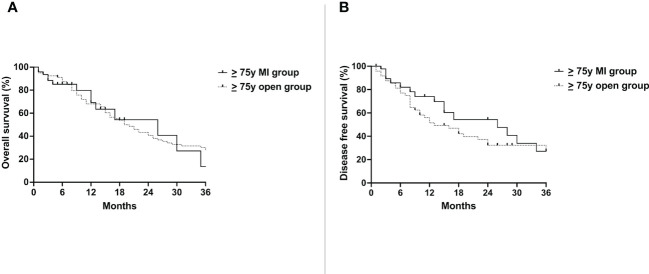
Overall survival **(A)** and disease free survival **(B)** of the ≥ 75y MI group vs. the ≥ 75y open group. Whole cohort.

No difference was detected in the OS of the ≥ 75y MI group (26 months) and the ≥ 75y open group (19 months)(p=0.84) (**Figure 2A**). The DFS was also similar between the two groups (26 months vs. 13 months, p=0.25) (**Figure 2B**).

## 4 Discussion

In this multicentric cohort study, the use of a minimally invasive approach improved the postoperative outcome of elderly patients undergoing Ivor Lewis esophagectomy by significantly reducing the rate of pulmonary complications, particularly of pneumonia and mucous plugging causing atelectasis. This translated into a significantly shorter hospital stay. Although not significantly, the rate of ICU re-admission and reoperation were also decreased after MIE/RAMIE. The results were confirmed at the one-to-one matched univariate analysis of the elderly group, moreover, similar outcomes were reported in the control group comprising younger patients.

Esophagectomy is a complex procedure, the rate of postoperative morbidity reported in the literature ranges widely between 20% and 80% (1, 2) while postoperative mortality ranges from 0% to 22% (1, 3). This seems to correspond to a higher postoperative morbidity and mortality rate in elderly patients compared to the younger ones (27). In a 2013 meta-analysis, Markar et al. reported an increased risk of pulmonary (21.8%) and cardiac complications (18.7%) after esophagectomy for patients > 70 years-old, with a 2 fold increase in the risk of postoperative death (7.8%) and a reduced cancer-related 5-year survival (21.2%) (28). Similarly, Schlottmann et al. reported that the predicted probability of mortality increased consistently across age (2.5% in 50 years, 5.4% in 70 years and 7% in 80) (29). These findings are understandable, since elderly patients represent a fragile subset often presenting in worse baseline clinical conditions and, consequently, with reduced reserves and capacity to endure major surgical procedures (1, 30). In our cohort, the ≥ 75y group presented with a significantly worse performance status and a higher comorbidity index compared to the control group. The overall in-hospital mortality of the elderly group was indeed higher, although not significantly, compared to the < 75y patients (4.4% vs. 1.9%, p = 0.09). However, this mortality rate doesn’t seem to be prohibitive, if we consider that the currently published benchmarks for mortality after esophagectomy performed in optimal conditions (i.e. in healthy patients and referral centers) range between 2.3% and 5.1% (1, 31).

Minimally invasive esophagectomy has been introduced in the last decades with the aim of reducing the surgical trauma, the complications and improving the quality of life after surgery. Currently, 4 randomized controlled trials comparing hybrid- (32), totally-minimally invasive esophagectomy (4, 33) and RAMIE (5) to open surgery demonstrated a reduction in overall postoperative complications and pulmonary complications. In this context, the use of a minimally invasive approach might seem particularly beneficial in the elderly population, which is more prone to the inherent morbidity of open esophagectomy. However, the matter is more controversial if we consider two factors. First, the reported outcomes of minimally invasive esophagectomy are not uniformly favorable in the published literature: the TIME trial showed an almost two-fold increase of the anastomotic leakage rate after MIE compared to the open approach (12% vs. 7%) (4). Similarly, in the large population-based study from the Dutch Upper Gastrointestinal Cancer Audit (DUCA) (34) the anastomotic leak and the reintervention rates were higher after MIE (21.2% vs. 15.5% and 28.2% vs. 21.1% respectively). This is particularly relevant considering that the leakage-related mortality has been reported to be up to 8.5 time higher in elderly patients compared to the younger ones (35). Second, several authors still found significantly higher pulmonary complications and mortality rates among elderly patients despite being operated without a trans-thoracic approach (35–37). Therefore, the actual benefit of using thoracoscopy for the thoracic phase might be limited. Taken together, these findings may question the benefit of using MIE or RAMIE in elderly patients.

Several studies compared the outcomes of minimally invasive esophagectomy between cohorts of elderly and non-elderly patients, reporting less cardiovascular complications (7), anastomotic leakages (8) and a reduced 90-day mortality (9) among younger patients. However, the literature directly comparing different surgical approaches for esophagectomy in the elderly population is somewhat limited. In a retrospective cohort study from 2015, Li et al. (10) analyzed the postoperative outcomes of 407 patients older than 70 years who underwent either MIE or open esophagectomy. After paired matching of 116 patients (58 pairs) the authors reported a significantly reduced rate of postoperative complications, particularly of pulmonary complications, with a shorter hospital stay and a reduced need for ICU readmission after MIE. In this series, however, 96.6% of patients in the MIE group underwent McKeown esophagectomy with cervical anastomosis, therefore the results are hardly applicable to the Western clinical practice, were intra-thoracic anastomoses are more frequently performed (38).

The effect of using a minimally invasive approach on the incidence of postoperative leakage after Ivor Lewis is still unclear (4, 34). Given the higher leakage-associated mortality among elderly patients (35), whether it is beneficial to perform MIE-Ivor Lewis in an elderly subject remains an unanswered question. To the best of our knowledge, our case series is one of the largest addressing this issue, by directly comparing the outcome of minimally invasive and open Ivor Lewis esophagectomy in the elderly.

In our study, the minimally invasive approach proved not only to be feasible in ≥ 75 years-old-patients, but also effective in improving the postoperative course by reducing postoperative complications. A significant reduction in pulmonary complications was the main determinant of the final outcome and this is coherent with the results reported in most of the currently available cohort studies (6) and the TIME trial (4). Even more interestingly, in our study more than half of the patients in the ≥ 75y MI group were operated using RAMIE (58.6%), thus confirming the feasibility and safety of this approach for healthy subjects. Prior to our study the evidence regarding the use of RAMIE in elderly subjects was rather limited considering that the mean age of the robotic-cohort in the ROBOT trial was 64 years (5).

The presence of a bias due to the *a priori* selection of elderly patients in better overall condition and performance status for MIE/RAMIE rather than open surgery might be a limit of this study. This might explain the significant differences in the clinical characteristics of elderly patients at presentation (cancer histology, location, and stage) that was detected in our cohort. Another issue might be the relatively small sample size of the ≥ 75y MI group compared to the open one. However, the fact that the results were confirmed after matching our cohort and that the control group showed a similar trend in the postoperative course seems to ascertain the benefit provided by the minimally invasive approach in our series.

The multicentric design of this study and the inclusion of both MIE and RAMIE cases might also be a limitation since technical differences between the two approaches and the two recruiting centers should be accounted for. However, we believe that this aspect had a limited impact on our results since the operative setting and the key surgical steps of the two procedures, particularly the end-to-side circular-stapled anastomosis, were practically identical and the same anastomotic technique was also used for the open-cases.

Finally, we decided to use a cutoff value of 75 years of age to define elderly patients, while other studies used other cutoffs, e.g. 70 years (10, 28). The definition of elderly patients is indeed rather variable in the literature. However, esophageal cancer is most frequently diagnosed among people aged 65 to 74 years, the median age at diagnosis being 68 years. Indeed, according to NIH data, the percentage of new cases is highest in the 65-to-74-year age group, reaching 33.3% (39).

For these reasons, considering the aim of the study, we believe it would’ve been misleading to use a lower cutoff value to define “elderly patients with esophageal cancer” (such as 60 or 70 years). Such a subgroup (> 60 years or > 70 years patients) wouldn’t in fact be representative of a group of elderly esophageal-cancer patients, but rather of the average-aged esophageal cancer patient.

Nevertheless, we obtained the same results reported in the study even conducting a separate analysis using > 70 years as a cutoff (see **Supplementary Material**).

## 5 Conclusions

The use of a minimally invasive approach for Ivor Lewis esophagectomy, including the robotic approach, is feasible and safe in elderly patients, even though this subset of patients usually presents in a worse clinical condition and with a poor performance status. Compared to open surgery, the improvement in terms of reduction of postoperative complications, particularly pulmonary complications, is comparable between elderly and younger patients. The surgical radicality and the incidence of procedure-specific complications (anastomotic leakage, conduit necrosis, chyle leak etc.) is comparable between the two procedures in both age-groups.

## Data availability statement

The raw data supporting the conclusions of this article can be made available by the authors under reasonable request to the corresponding author at lucia.moletta@unipd.it.

## Ethics statement

Ethical review and approval was not required for the study on human participants in accordance with the local legislation and institutional requirements. Written informed consent for participation was not required for this study in accordance with the national legislation and the institutional requirements.

## Author contributions

Study conception and design: GC, EU, LM, EH, EP, GZ, ET, FB, HL, MV, and PG; Acquisition of data: GC, EU, AS, LM, EH, LP, RS, EP, GZ, ET, and FB; Analysis and interpretation of data: GC, EU, LM, EH, LP, RS, ET, FB, HL, MV, and PG; Drafting of manuscript: GC, EU, AS, LM, and EH; Critical revision: LP, RS, EP, GZ, ET, FB, HL, MV, and PG. All authors contributed to the article and approved the submitted version.
