# Supplementary material for: Minimally invasive Ivor Lewis esophagectomy in the elderly patient: a multicenter retrospective matched-cohort study
Source: Front Oncol. 2023 May 12;13:1104109. doi: 10.3389/fonc.2023.1104109 (PMC10213659; doi:10.3389/fonc.2023.1104109)
Supplement: Supplementary file 1 [file Table_1.docx]

| **Table 1. Clinical characteristic of the studied population using the 70-yearcutoff value** | | | |
| --- | --- | --- | --- |
| **Variable** | **< 70y group (N=407)** | **> 70y group (N=180)** | **p** |
| Median age (years) | 58 (52-64) | 74 (71-76) | 0.004 |
| Sex (N, %) |  |  |  |
| Male | 338 (83) | 137 (76.1) | 0.04 |
| Female | 69 (17) | 43 (23.9) |  |
| Height (m) | 1.75 (1.69-1.78) | 1.72 (1.65-1.77) | 0.03 |
| Weight (kg) | 77 (68-90) | 73 (63-88) | 0.10 |
| ASA score (N, %) |  |  |  |
| 1 | 5 (1.2) | 1 (0.6) | < 0.0001 |
| 2 | 211 (51.8) | 52 (28.9) |  |
| 3 | 185 (45.5) | 107 (59.4) |  |
| 4 | 6 (1.5) | 20 (11.1) |  |
| Karnofsky performance status (N, %)* |  |  |  |
| 100-90 | 363 (89.2) | 142 (78.9) | 0.003 |
| 80-70 | 42 (10.3) | 35 (19.4) |  |
| 60-50 | 2 (0.5) | 3 (1.7) |  |
| Charlson’s Comorbidity Index (CCI) | 5 (4-6) | 6 (5-7) | 0.01 |
| Cancer histology (N, %) |  |  |  |
| ESCC | 73 (17.9) | 55 (30.6) | 0.0006 |
| EAC | 334 (82.1) | 125 (69.4) |  |
| Cancer location (N, %) |  |  |  |
| Thoracic esophagus | 51 (12.5) | 42 (23.3) | 0.004 |
| Siewert 1 | 201 (49.4) | 82 (45.6) |  |
| Siewert 2 | 155 (38.1) | 56 (31.1) |  |
| cTNM Staging |  |  |  |
| Stage 1 | 37 (9.2) | 15 (8.3) | 0.97 |
| Stage 2 | 84 (20.6) | 35 (19.4) |  |
| Stage 3 | 259 (63.6) | 118 (65.6) |  |
| Stage 4 | 27 (6.6) | 12 (6.7) |  |
| Perioperative treatment (N, %) |  |  |  |
| None | 64 (15.7) | 99 (55) | < 0.0001 |
| Chemotherapy | 162 (39.8) | 39 (21.7) |  |
| Chemoradiotherapy | 180 (44.2) | 39 (21.7) |  |
| Radiotherapy | 1 (0.3) | 3 (1.6) |  |
| Surgical approach (N, %) |  |  |  |
| Open | 171 (42) | 108 (60) | 0.0003 |
| Laparoscopic/Thoracoscopic (MIE) | 90 (22.1) | 29 (16.1) |  |
| Fully Robotic (RAMIE) | 146 (35.9) | 43 (23.9) |  |

| **Table 2. Clinical characteristics of the analyzed subgroups using the 70-yearcutoff value** | | | | | | |
| --- | --- | --- | --- | --- | --- | --- |
| **Variable** | **< 70y MI group (N=236)** | **< 70y open group (N=171)** | **p** | **> 70y MI group (N=72)** | **> 70y open group (N=108)** | **p** |
| Median age (years) | 57 (53-68) | 60 (55-66) | 0.30 | 72 (74-80) | 75 (70-77) | 0.43 |
| Sex (N, %) |  |  |  |  |  |  |
| Male | 193 (81.8) | 145 (84.8) | 0.42 | 61 (84.7) | 76 (70.4) | **0.03** |
| Female | 43 (18.2) | 26 (15.2) |  | 11 (15.3) | 32 (29.6) |  |
| Height (m) | 1.75  (1.63-1.77) | 1.77  (1.70-1.82) | 0.54 | 1.72  (1.65-1.78) | 1.70  (1.59-1.76) | 0.35 |
| Weight (kg) | 78 (69.5-89) | 79 (67-91) | 0.31 | 73 (62-87) | 72 (53-81) | 0.68 |
| ASA score (N, %) |  |  |  |  |  |  |
| 1 | 3 (1.3) | 2 (1.2) | 0.20 | 0 | 1 (0.9) | 0.88 |
| 2 | 113 (47.9) | 98 (57.3) |  | 21 (29.2) | 31 (28.7) |  |
| 3 | 115 (48.7) | 70 (40.9) |  | 43 (59.7) | 64 (59.3) |  |
| 4 | 5 (2.1) | 1 (0.6) |  | 8 (11.1) | 12 (11.1) |  |
| Karnofsky performance status (N, %) |  |  |  |  |  |  |
| 100-90 | 208 (88.1) | 155 (90.6) | 0.41 | 56 (77.8) | 86 (79.6) | 0.63 |
| 80-70 | 26 (11) | 16 (9.4) |  | 14 (19.4) | 21 (19.4) |  |
| 60-50 | 2 (0.9) | 0 |  | 2 (2.8) | 1 (0.9) |  |
| Charlson’s Comorbidity Index (CCI) | 5 (3.5-6) | 4 (3-5) | 0.16 | 5 (4-7) | 6 (5-7) | 0.31 |
| Cancer histology (N, %) |  |  |  |  |  |  |
| ESCC | 49 (20.8) | 24 (14) | 0.71 | 22 (30.6) | 33 (30.6) | 0.37 |
| EAC | 187 (79.2) | 147 (86) |  | 50 (69.4) | 75 (69.4) |  |
| Cancer location (N, %) |  |  |  |  |  |  |
| Thoracic esophagus | 29 (12.3) | 22 (12.9) | 0.41 | 12 (16.7) | 30 (27.8) | 0.19 |
| Siewert 1 | 123 (52.1) | 78 (45.6) |  | 34 (47.2) | 48 (44.4) |  |
| Siewert 2 | 84 (35.6) | 71 (41.5) |  | 26 (36.1) | 30 (27.8) |  |
| cTNM Staging |  |  |  |  |  |  |
| Stage 1 | 27 (11.4) | 10 (5.8) | 0.23 | 8 (11.1) | 7 (6.5) | 0.61 |
| Stage 2 | 48 (20.3) | 36 (21.1) |  | 12 (16.7) | 23 (21.3) |  |
| Stage 3 | 144 (61.1) | 115 (67.3) |  | 48 (66.7) | 70 (64.8) |  |
| Stage 4 | 17 (7.2) | 10 (5.8) |  | 4 (5.5) | 8 (7.4) |  |
| Perioperative treatment (N, %) |  |  |  |  |  |  |
| None | 41 (17.4) | 23 (13.5) | 0.26 | 33 (45.8) | 66 (61.1) | 0.21 |
| Chemotherapy | 98 (41.5) | 64 (37.4) |  | 18 (25) | 21 (19.4) |  |
| Chemoradiotherapy | 97 (41.1) | 83 (48.5) |  | 20 (27.8) | 19 (17.6) |  |
| Radiotherapy | 0 | 1 (0.6) |  | 1 (1.4) | 2 (1.9) |  |
| Surgical approach (N, %) |  |  |  |  |  |  |
| Laparoscopic/Thoracoscopic (MIE) | 90 (38.1) | - | - | 29 (40.3) | - | - |
| Fully Robotic (RAMIE) | 146 (61.9) | - |  | 43 (59.7) | - |  |
| Operative time (min) | 326  (283-379) | 280  (241-329) | 0.03 | 341  (309-377) | 299  (240-336) | 0.02 |

| **Table 3. Surgical outcomes of the analyzed subgroups using the 70-yearcutoff value** | | | | | | |
| --- | --- | --- | --- | --- | --- | --- |
| **Variable** | **< 70y MI group (N=236)** | **< 70y open group (N=171)** | **p** | **> 70y MI group (N=72)** | **> 70y open group (N=108)** | **p** |
| Surgical radicality (N, %) |  |  |  |  |  |  |
| R0 | 232 (98.3) | 167 (97.7) | 0.64 | 69 (95.8) | 105 (97.2) | 0.61 |
| R1 | 4 (1.7) | 4 (2.3) |  | 3 (4.2) | 3 (2.8) |  |
| Harvested lymph nodes  (N, IQR) | 30 (22-41) | 29 (27-33) | 0.51 | 27 (18-33) | 26 (16-32) | 0.44 |
| Metastatic lymph nodes  (N, IQR) | 2 (0-3) | 2 (0-2) | 0.31 | 2 (0-3) | 1 (0-4) | 0.48 |
| Intraoperative complications (N, %) | 8 (3.4) | 5 (2.9) | 0.79 | 4 (5.6) | 5 (4.6) | 0.78 |
| Postoperative morbidity  (N, %) | 73 (30.9) | 73 (42.7) | 0.02 | 28 (38.9) | 68 (62.9) | 0.002 |
| Anastomotic leakage (N, %) | 24 (10.2) | 11 (6.4) | 0.18 | 9 (12.5) | 19 (17.6) | 0.67 |
| Grade 1 | 1 (0.4) | 0 | 0.74 | 2 (2.8) | 2 (1.8) | 0.60 |
| Grade 2 | 20 (8.5) | 10 (5.8) |  | 5 (6.9) | 14 (12.9) |  |
| Grade 3 | 3 (1.3) | 1 (0.6) |  | 2 (2.8) | 3 (2.8) |  |
| Conduit necrosis (N, %) | 1 (0.4) | 2 (1.2) | 0.57 | 1 (1.4) | 2 (1.9) | 0.99 |
| Chyle leak (N, %) | 7 (2.9) | 3 (1.7) | 0.53 | 4 (5.6) | 4 (3.7) | 0.71 |
| Vocal cord palsy (N, %) | 3 (1.3) | 2 (1.2) | 0.99 | 0 | 4 (3.7) | 0.15 |
| Hemothorax (N,%) | 7 (2.9) | 9 (5.2) | 0.30 | 2 (2.8) | 9 (8.3) | 0.20 |
| Pulmonary complications (N,%)* | 51 (21.6) | 60 (35.1) | 0.003 | 23 (31.9) | 62 (57.4) | 0.0008 |
| Pneumonia | 14 (5.9) | 20 (11.7) | 0.04 | 6 (8.3) | 22 (20.4) | 0.03 |
| Atelectasis mucous plugging requiring bronchoscopy | 16 (6.8) | 19 (11.1) | 0.12 | 8 (11.1) | 26 (24.1) | 0.03 |
| Pneumothorax | 7 (2.9) | 7 (4.1) | 0.59 | 1 (1.4) | 4 (3.7) | 0.65 |
| Pleural effusion requiring drainage | 6 (2.5) | 5 (2.9) | 0.81 | 3 (4.2) | 5 (4.6) | 0.99 |
| Respiratory failure requiring reintubation | 5 (2.1) | 6 (3.5) | 0.39 | 3 (4.2) | 4 (3.7) | 0.99 |
| ARDS | 3 (1.3) | 3 (1.7) | 0.69 | 0 | 1 (0.9) | 0.99 |
| Cardiac complications (N,%)* | 17 (7.2) | 14 (8.2) | 0.71 | 8 (11.1) | 13 (12) | 0.85 |
| Atrial dysrhythmia  requiring treatment | 10 (4.2) | 11 (6.4) | 0.32 | 5 (6.9) | 6 (5.6) | 0.70 |
| Congestive heart failure requiring treatment | 7 (2.9) | 3 (1.7) | 0.43 | 3 (4.2) | 6 (5.6) | 0.67 |
| Myocardial infarction | 0 | 0 | 0.99 | 0 | 1 (0.9) | 0.99 |
| Gastrointestinal complications (N,%)* | 7 (2.9) | 9 (5.2) | 0.24 | 1 (1.4) | 4 (3.7) | 0.35 |
| *Clostridium difficile* infection | 1 (0.4) | 2 (1.2) | 0.38 | 1 (1.4) | 3 (2.8) | 0.54 |
| Liver dysfunction | 1 (0.4) | 1 (0.6) | 0.82 | 0 | 1 (0.9) | 0.99 |
| Sepsis | 5 (2.1) | 5 (2.9) | 0.60 | 2 (2.8) | 12 (11.1) | 0.04 |
| Complications: severity^b^  (N, %) |  |  |  |  |  |  |
| 1 | 6 (2.5) | 8 (4.7) | 0.80 | 1 (1.4) | 3 (2.8) | 0.92 |
| 2 | 23 (9.7) | 18 (10.5) |  | 5 (6.9) | 12 (11.1) |  |
| 3a | 17 (7.2) | 16 (9.4) |  | 8 (11.1) | 26 (24.1) |  |
| 3b | 10 (4.2) | 11 (6.4) |  | 4 (5.6) | 9 (8.3) |  |
| 4 | 12 (5.1) | 17 (9.9) |  | 8 (11.1) | 13 (12) |  |
| 5 | 5 (2.1) | 3 (1.7) |  | 2 (2.8) | 5 (4.6) |  |
| Need for reoperation (N, %) | 13 (5.5) | 15 (8.8) | 0.20 | 6 (8.3) | 12 (11.1) | 0.54 |
| ICU readmission (N, %) | 16 (6.8) | 18 (10.5) | 0.18 | 9 (12.5) | 20 (18.5) | 0.28 |
| Lenght of hospital stay (days)(N, IQR) | 12 (9-15) | 12 (10-16) | 0.19 | 13 (10-18) | 17 (13-25) | 0.04 |
| In-hospital mortality (N, %) | 5 (2.1) | 3 (1.7) | 0.99 | 2 (2.8) | 5 (4.6) | 0.70 |

*The most severe complication for each category is indicated.
